# Surgical Outcomes After Frontalis Suspension Using Expanded Polytetrafluoroethylene Sling for Congenital Ptosis

**DOI:** 10.7759/cureus.49020

**Published:** 2023-11-18

**Authors:** Benjamin K Ghiam, Robin C Su, Faruk Orge

**Affiliations:** 1 Department of Ophthalmology, Roski Eye Institute, University of Southern California, Los Angeles, USA; 2 Department of Ophthalmology and Visual Sciences, University Hospitals Eye Institute, Case Western Reserve University, Cleveland, USA; 3 Department of Ophthalmology and Visual Sciences, University Hospitals Rainbow Babies and Children's Hospital, Case Western Reserve University, Cleveland, USA

**Keywords:** oculoplastic surgery, pediatric ophthalmology, expanded polytetrafluoroethylene sling, frontalis suspension, congenital ptosis

## Abstract

Purpose

The purpose of the study is to assess short- and long-term functional outcomes after frontalis suspension using expanded polytetrafluoroethylene (ePTFE) sling for congenital ptosis repair.

Methods

A retrospective, observational case review was conducted on pediatric patients who underwent frontalis suspension using ePTFE sling from 2008 to 2020. Functional success was assessed by lid height, lid symmetry, and parental satisfaction with the cosmetic outcome. Clinical course and long-term functional outcomes after surgery were assessed.

Results

Twenty-one cases met the inclusion criteria and were assessed. The follow-up time ranged from 13 months to 11 years (mean: six years). Functional success after one surgery was 62% at early and late postoperative periods. Six of 21 cases (29%) required revisional surgery in the early postoperative period due to undercorrection. Three cases (14%) were complicated by infection and/or granuloma formation. There were no cases of ptosis recurrence in the long term if success was seen in the early postoperative period.

Conclusion

ePTFE slings remain an excellent option for severe congenital ptosis repair with frontalis sling, demonstrating long-term functional success, with satisfactory lid symmetry and acceptable cosmetic outcome. This is of important consideration in patients younger than three years of age, where autogenous materials may not be recommended. The need for early revisional surgery for undercorrection is not uncommon. The current authors also demonstrate a low but considerable risk for infection and/or granuloma formation.

## Introduction

The diagnosis of congenital ptosis is considered if the ptosis is present at birth or diagnosed within the first year. Severe forms have been attributed to the hypoplasia of the levator palpebrae superioris muscle or tendon, resulting in poor levator function; it is often unilateral in about 70% of cases and may be isolated or associated with other ocular and/or systemic conditions [1,2]. Children with congenital ptosis may suffer from obstructed visual fields or may experience significant induced refractive error, which may lead to amblyopia. For these reasons, providers have advocated for pursuing early surgical intervention to prevent the development of these often irreversible visual consequences [3].

The evolution of surgical techniques has changed considerably over decades, owing to the need for long-lasting functional outcomes that not only minimize visual deprivation and induced refractive error but also prevent overcorrection and subsequent exposure keratopathy, combined with the challenge of producing cosmetically acceptable results, with the preservation of the normal eyelid crease and eyelid contour. Thus, it is not surprising that the correction of congenital ptosis has been described as one of the most difficult challenges ophthalmologists face [3]. Many surgical techniques have been described, including frontalis sling, levator resection, Whitnall's ligament sling, and Muller's muscle resection, each with its own advantages and disadvantages, with considerations that may depend on the degree of ptosis, levator function, and surgeon preference [3].

The most common surgical approach to patients with congenital ptosis with severe ptosis and poor levator function (<4 mm) is the frontalis sling [4]. The technique consists of forming a direct connection between the tarsal plate of the upper eyelid and the frontalis muscle. This allows the eyelid to be elevated by the use of the frontalis muscle. This approach has had varying outcomes, with much of the functional and cosmetic outcomes related to the choice of sling material and the position of the material within the eyelid [5].

Materials can generally be divided into three groups: synthetic, autograft fascia lata, or banked allograft fascia lata. Many different types of synthetic materials have been investigated, including collagen, silicone, polypropylene, silk, nylon, polyester, steel, and expanded polytetrafluoroethylene (ePTFE) [5]. Ben Simon et al. investigated and compared the various surgical materials that may be used in ptosis repair, citing an overall rate of recurrence of 26% at 20 months postoperatively, with polytetrafluoroethylene (PTFE) slings producing the lowest incidence of recurrence (15%) and demonstrating long postoperative success [5]. Common disadvantages to all frontalis sling approaches include the risk of the lagophthalmos of the eyelid on down gaze, scarring, unsatisfactory geometric tenting of the pretarsal and preseptal skin, loss of the eyelid crease, and poor tarso-corneal interface seen upon brow elevation and down gaze [5-7].

The current study assesses long-term functional outcomes of frontalis suspension using ePTFE sling material in pediatric patients with severe congenital ptosis with poor levator function (<4 mm). Surgical results were assessed in the early and late postoperative stages. The outcomes of lid height (defined as margin-to-reflex distance {MRD}), lid symmetry, and parental satisfaction with cosmetic outcomes were analyzed.

## Materials and methods

Written and informed consent was taken from all patients. This study was approved by the Rainbow Hospital Institutional Ethics Committee and Institutional Review Board (approval number: HRP-503DATA; date: 05/2021). All procedures performed in our study involving human participants were in accordance with the 1964 Helsinki Declaration and its later amendments.

A retrospective chart review was performed of pediatric patients with severe congenital ptosis (levator function: <4 mm) who underwent unilateral or bilateral frontalis suspension using expanded polytetrafluoroethylene (ePTFE) (Ptose-up, FCI Ophthalmics, Pembroke, MA) from 2009 to 2020, under the care of one surgeon. In all patients, the indications for surgery were a levator palpebrae superioris function of less than 4 mm and a prolonged significant occlusion of the visual axis by the ptotic lid. The study excluded patients with less than 12 months of follow-up. Postoperative assessments were performed by the primary surgeon at one week, one month, and six months and at appropriate individualized intervals thereafter. The study complied with the policies of the local Institutional Review Board. The data retrieved includes age, gender, diagnosis, pre- and postoperative margin-to-reflex distance (MRD), preoperative levator palpebrae superioris function, palpebral fissure height, and postoperative parental satisfaction with cosmesis. The MRD was measured with the eyelid(s) "at rest," that is, when the patient was not actively contracting their frontalis muscles.

Functional success was defined by fulfilling the following three criteria, without serious complications such as infection and keratopathy resulting from entropion or exposure: 1) satisfactory lid height (defined as MRD of at least 3 mm), 2) satisfactory lid symmetry (<2 mm asymmetry in MRD), and 3) parental satisfaction with the cosmetic outcome.

Recurrence was defined by a drop in lid height of >3 mm from the initial postoperative level. Postoperative eyelid or ocular complications were also noted. These criteria are adapted from the criteria described by Manners et al. [8].

Surgical technique

**Figure 1:**
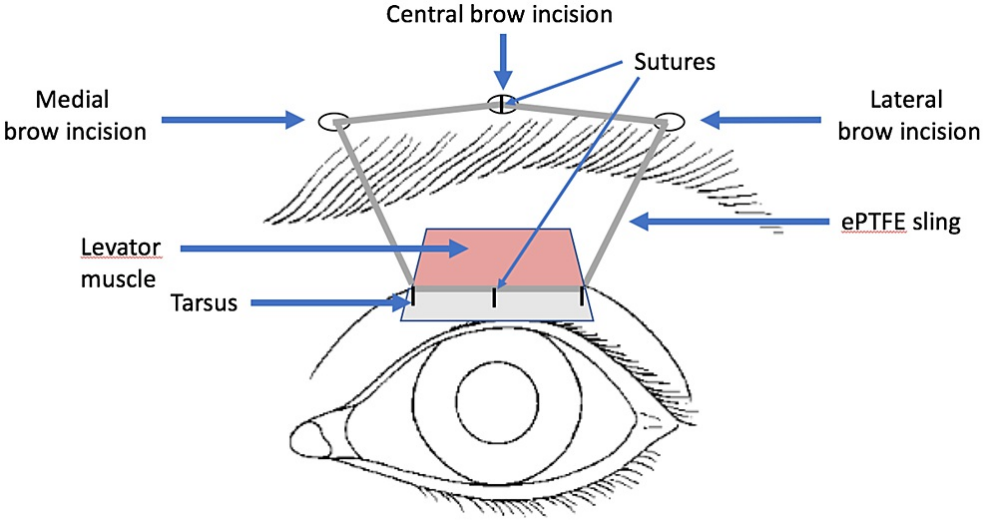
Diagrammatic illustration of the ePTFE sling technique. This diagram was created by the authors of this manuscript. ePTFE: expanded polytetrafluoroethylene

All surgical procedures were performed under general anesthesia. Figure 1 demonstrates the ePTFE sling technique used. After antiseptic preparation, digital pressure was then applied for hemostasis, and a number 11 blade was then used to make three stab incisions immediately superior to the brow, one at the level of the lateral canthus, one at the level of the medial canthus, and the third centrally located. The number 11 blade was then used to make a lid crease incision along the marked site. The tissue was carefully dissected to expose the tarsal plate. Gentle cautery was used for hemostasis. The ePTFE sling was attached to the tarsal plate using three interrupted 6-0 Vicryl sutures. A Wright needle was then used to tunnel from the lid crease incision to the medial brow incision. The needle was then used to carry the sling to the medial brow incision. The same was done temporally. The Wright needle was then used to tunnel from the medial brow incision to the center of the brow incision, and the needle was used to carry the sling to the central brow incision. The same was done temporally. The two ends of the sling material were tied while obtaining an accurate lid opening. The knot was further secured/reinforced with the addition of 5-0 Mersilene suture. All wounds were then closed with interrupted 6-0 chromic gut sutures, and the surgical procedure was complete. Topical ophthalmic antibiotic ointment was instilled and provided to the patient. Patients were monitored for one week, one month, and six months following the procedure. Thereafter, the patients were evaluated at regular intervals as indicated for their postoperative course.

## Results

Of the 28 total cases with severe congenital ptosis (levator function: <4 mm) that underwent frontalis sling suspension with ePTFE sling material, 21 cases had follow-up beyond six months. Table 1 summarizes the demographics of these patients. There were 10 males and 11 females, ranging from 0.57 to 12.75 years of age, with a mean age of 2.3 years (standard deviation: 2.2 years). The mean follow-up time was about 5.9 years, with a range of 1.1 years to 11 years (standard deviation: 3.9 years).

**Table 1:** Baseline demographics of patients included in the study (N=21). The data has been represented by mean±SD. SD: standard deviation

Number of patients	21
Gender (male/female)	10/11
Age at surgery (years)	
Mean±SD	2.3±2.2
Range	0.57-12.75
Follow-up (years)	
Mean±SD	5.9±3.9
Range	1.1-11

**Figure 2:**
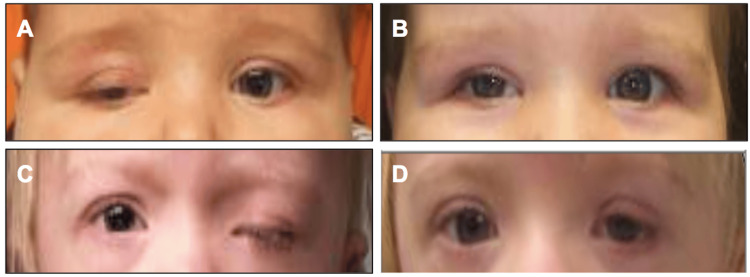
Before and after images of congenital ptosis repair with ePTFE sling suspension. Images used with permission obtained from the patients' parents or legal guardians. (A) The image of the example patient number 1 before congenital ptosis repair. (B) The image of the example patient number 1 after congenital ptosis repair with ePTFE sling suspension. (C) The image of the example patient number 2 before congenital ptosis repair. (D) The image of the example patient number 2 after congenital ptosis repair with ePTFE sling suspension. ePTFE: expanded polytetrafluoroethylene

Table 2 summarizes the outcomes of the cases. Functional success was assessed in the early and late postoperative periods. Thirteen of 21 cases (62%) demonstrate early and late postoperative success, without requiring any surgical revision (Figure 2). Four cases demonstrated early postoperative undercorrection at postoperative month 1 requiring revision; among those cases, one demonstrated long-term success following a single revision procedure, and the other three required two additional revision procedures with subsequent long-term success. Two cases demonstrated a slipped sling within one month postoperatively, requiring revision with subsequent long-term success. Three cases were complicated by infection and/or granuloma formation, one case resolved with oral antibiotics, one case required surgical intervention and sling revision with subsequent success, and one required resection without additional attempts for repair. One case demonstrated mild overcorrection with corneal exposure at postoperative month 1, which self-resolved on subsequent follow-up.

**Table 2:** Functional success and complications after ePTFE sling suspension (N=21). The data has been represented as N (%) of the 21 subjects. ePTFE: expanded polytetrafluoroethylene

	Eyelid cases (n=21)
Functional success	13 (62%)
Complications	
Slipped sling (one additional revision surgery)	2 (10%)
Infection	3 (14%)
Undercorrection	4 (20%)
One additional surgery	1 (5%)
Two additional surgeries	3 (14%)
Overcorrection (self-resolving)	1 (5%)

## Discussion

There has been some debate over sling material in ptosis repair [5,7,9,10]. Many providers elect to use autogenous fascia lata because it has been shown to result in lower recurrence (about 5%) and lower risk of infection and/or granuloma formation [5-7,11]. Some providers, such as Crawford, believe that an autogenous fascia lata material is not a good option in patients younger than three years old, stating that this tissue can be difficult to harvest and that when it is able to be successfully harvested, the amount of material is frequently not sufficient for successful operation [11]. Other providers, such as Evereklioglu, have reported good functional and aesthetic results with autogenous fascia lata with recent advanced surgical techniques [12]. Though autogenous fascia lata may provide good results, this technique inevitably requires a second surgical site, which may lead to other unnecessary complications and recovery for the patient. Depending on the comfort level of the ophthalmologist, this technique may also require coordination with other non-ophthalmic surgeons, adding to the cost and complexity of the surgery planning.

Because of the early and irreversible risks of amblyopia in cases of severe congenital ptosis (levator function: <4 mm), surgeons have been compelled to find alternative materials in cases where intervention is advised in patients younger than three years old. Banked allogenic fascia lata is an alternative option, though studies suggest an increased recurrence rate, up to 51.5%, and increased inflammatory reactions [7,11]. Synthetic materials have also been enticing to many surgeons, as these are more readily available and do not carry the risk of transmitting infectious diseases [7,13]. Additionally, despite the fact that autogenous fascia has better biocompatibility than alloplastic materials, similar functional and cosmetic outcomes are often achieved with alloplastic materials [5].

In the late 1980s, oculoplastic surgeons began investigating the use of expanded polytetrafluoroethylene (ePTFE), an inert, nonantigenic, and biocompatible synthetic material that had been previously used in vascular and abdominal surgeries. Surgeons employ this material because it is easily suturable and has great bio-integration by means of fibroblastic ingrowth [10,11]. While many believe that suspension with synthetic materials (such as Prolene and nylon) should be considered a temporary procedure as these materials are often associated with a high risk of recurrence, many studies have demonstrated long-term success in ePTFE [7].

Previous studies that have studied the outcomes of ePTFE have demonstrated excellent recurrence rates. Steinkogler et al. demonstrated one case of recurrence requiring surgical repair among 37 total cases that were followed up for an average of three years and only one case of "rejection" requiring surgical removal [14]. Other studies have similarly demonstrated no recurrent ptosis in patients up to 18 months of follow-up [7].

The current study confirms that the use of ePTFE for the correction of congenital ptosis in children results in good long-term functional outcomes and is a good alternative to ptosis repair. Among the 21 included cases, patients were followed up well beyond 12 months, with a mean follow-up time of about six years. Sixty-two percent of cases did not require any revisions since the last follow-up and demonstrated short- and long-term functional success from one procedure. Additionally, there were no cases of ptosis recurrence in the long term if success was seen in the early postoperative period (within six months postoperatively). All cases that required revisional surgery presented in the early postoperative period with undercorrection, of which there were six of 21 cases (29%). Two of these included "slipped" slings requiring surgical revision.

In the two cases that demonstrated "slipped" slings at early postoperative follow-up, both cases featured 2 mm ePTFE sling. During the subsequent revisional procedure, the slings were noted to have been torn; thus, a 3 mm-wide sling was placed, ultimately demonstrating short- and long-term postoperative success. In three of the remaining four cases that demonstrated early postoperative undercorrection, these children were noted to have developmental comorbidities that compromised postoperative care such that these children were noted to be chronic "headbangers," with family reporting frequent repetitive head hitting. These three patients required two revisional procedures, ultimately necessitating two slings in the final procedure, which proved stable at long-term follow-up.

The current authors found three cases that were complicated by infection and/or granuloma formation (14%). One resolved with oral antibiotics, one required surgical intervention and sling revision, and one required sling resection without additional attempts for repair. Of note, the patient in which the sling was removed without further attempts at repair had been a known methicillin-resistant *Staphylococcus aureus* (MRSA) carrier, with a history of MRSA infections in other parts of the body. Other studies have demonstrated variable rates of complications due to infection and/or granuloma formation. Wasserman et al. described an increased rate of infection and/or granuloma formation of more than fourfold with ePTFE slings [7]. However, several other studies have suggested that ePTFE can be used safely with good biocompatibility [15-18]. In their review of 90 total ptotic eyelids repaired with ePTFE frontalis suspension, Wei and Liao found that five out of 90 eyelids experienced infection and/or granuloma formation [15]. The current authors speculate that the granulomatous reaction may be due to a reaction against the drape material that became adhered to the sling during intraoperative handling. The current surgeon made a conscious effort to closely inspect, clean, and express this material with smooth tier forceps, to make sure no foreign material was apparent on the sling before insertion.

Additional complications observed by the current authors include one case of early postoperative corneal exposure, which self-resolved on subsequent follow-up.

There are several limitations to the current study. This includes the limited power of the study, such that only 28 cases were followed up beyond the 12-month cutoff. Additionally, the assessment of cosmetic outcomes is limited to encounter notes that document MRD values and subjective assessments of symmetry and parental satisfaction with outcomes. This limitation could have been minimized with the use of routine photography on follow-up appointments, with independent observers objectively measuring symmetry and assessing lid contour. There is additional bias due to the fact that follow-up examinations were conducted by the performing surgeon and not by an independent observer.

## Conclusions

In conclusion, the current authors believe that ePTFE slings remain a viable option for severe congenital ptosis repair with frontalis sling, demonstrating long-term functional success, with satisfactory lid symmetry and acceptable cosmetic outcome. While recent advances have been made to autogenous fascia lata techniques, options in patients younger than three years of age still remain limited, and ePTFE slings provide a good option. The current authors have found that the need for revisional surgery with ePTFE slings is not uncommon, however, and is in most cases indicated in the early postoperative period in the setting of undercorrection. The current authors also demonstrate a low but considerable risk for infection and/or granuloma formation.
